# Gene silencing of HIF-2α disrupts glioblastoma stem cell phenotype

**DOI:** 10.20517/cdr.2019.96

**Published:** 2020-03-11

**Authors:** Leora M. Nusblat, Shaili Tanna, Charles M. Roth

**Affiliations:** 1 Department of Biomedical Engineering, Rutgers, The State University of New Jersey, Piscataway, NJ 08854, USA.; 2 Department of Chemical and Biochemical Engineering, Rutgers, The State University of New Jersey, Piscataway, NJ 08854, USA.

**Keywords:** Glioblastoma multiforme, cancer stem cell, short interfering RNA, tumor hypoxia

## Abstract

**Aim:**

Improved treatment strategies are desperately needed for eradicating cancer stem cells (CSCs), which drive malignancy and recurrence in glioblastoma multiforme. Hypoxic regions within the tumor microenvironment help maintain and promote the proliferation of CSCs. Here, we explored the effects of silencing hypoxia inducible factor-2α (HIF-2α) because of its specificity for CSCs within the hypoxic environment.

**Methods:**

Cancer stem cell neurospheres were formed by enriching from both the glioblastoma cell line U87 and from brain tumor stem cells isolated directly from human brain tumors. Silencing of human HIF-2α was performed using both commercial and in-house transfection of a validated short interfering RNA, with all results compared to an established non-silencing control short interfering RNA. Silencing of HIF-2α was established by Western blotting, and phenotypic effects were assayed by cell migration assays, cell viability measurements, and immunofluorescence staining of differentiation markers.

**Results:**

Transfection with either our previously reported pH-sensitive, cationic amphiphilic macromolecule-based delivery system or Lipofectamine was similarly effective in silencing HIF-2α. The chemotherapeutic resistance and neurosphere formation were reduced when HIF-2α was silenced. Migratory capacities in the presence of macrophage conditioned media were modulated. HIF-2α silencing was complementary to temozolomide treatment in producing phenotypic rather than cytotoxic effects.

**Conclusion:**

HIF-2α silencing under hypoxia inhibited CSC phenotypes while promoting differentiated cell phenotypes and is complementary to existing DNA alkylating treatments in inhibiting glioma CSC activity.

## INTRODUCTION

Similar to most solid tumors, glioblastoma multiforme contains avascular regions that result in decreased oxygen availability within the tumor tissue. These regions of hypoxia are particularly apparent in gliomas where angiogenesis tends to be highly disorganized and where local oxygen levels drop well below physiological levels^[1]^. Hypoxia is believed to maintain stem cells in tumors, in part by preventing their differentiation. Disrupting these hypoxic regions may provide a route to cancer stem cell (CSC) targeting^[2]^, which has been explored through strategies such as “vascular normalization”^[3,4]^. Instead of targeting the hypoxic niche itself, a challenging feat, a better approach may be to target the signaling response to hypoxic conditions, mediated through hypoxia inducible factors (HIFs). HIFs are heterodimers, with alpha and beta subunits, that function as transcription factors. Under normoxic conditions, HIF is complexed to the von Hippel Lindau tumor suppressor protein (pVHL), resulting in its ubiquitination and degradation in the proteasome. In the presence of hypoxia, pVHL dissociates from HIF, causing the latter to be stabilized and translocated to the nucleus, where it dimerizes with HIFb and binds to hypoxia-response elements, promoting transcription of target proteins^[5]^.

Hypoxia is an important factor in a tumor model since it is reflective of the tumor microenvironment where CSCs are enriched^[6]^. Under hypoxic conditions, HIFs are upregulated. Both HIF-1 and HIF-2 isoforms are expressed in gliomas and their roles seem to be overlapping^[7]^. Both HIF-1 and HIF-2 alpha and beta complexes bind hypoxia-response elements in the promoters of many genes, such as *VEGF*, in order to upregulate them in response to hypoxia. However, while HIF-1α and HIF-2α are 75% homologous, they have several notable differences in function. In particular, HIF-2α is highly expressed in CSCs in multiple cancers^[8]^. HIF-2α enhances the expression of genes involved in maintaining the stem-like properties of these cells, and it is specifically overexpressed in CSCs, enhancing proliferation and undifferentiated markers such as nestin and CD133^[9]^. HIF-2α also plays a role in metastasis by promoting angiogenesis. Both HIF-1α and HIF-2α are expressed in cell culture and spheroid models of glioblastoma including by U87 cells^[9]^. HIF-1α is induced rapidly in response to extreme hypoxia levels, ~1% O_2_, whereas HIF-2α is induced later, but, in response to moderate hypoxia levels, as high as 5% O_2_, its expression is sustained^[10]^. Furthermore, HIF-2α expression has not been detected in normal human macrophages or in non-stem tumor cells. Thus, HIF-2α is an attractive target since moderate hypoxia induces HIF-2α exclusively in CSCs, while HIF-1α is induced in both CSCs and non-stem tumor cells^[10,11]^.

The tumor microenvironment of glioblastoma multiforme is a complex tissue of cells, including astrocytes, macrophages, pericytes, fibroblasts, and endothelial cells. Macrophages play a crucial role in the immune response. However, due to signaling by CSCs, macrophages undergo a switch towards an immunosuppressive state, promoting angiogenesis, reducing phagocytosis, and inhibiting T-cell proliferation^[12]^. Some groups have reported an immunosuppressive role of HIF-2α in tumor associated macrophages^[13]^. Although both HIF-1α and HIF-2α are expressed in macrophages, HIF-2α accumulation in tumor associated macrophages is correlated with high tumor vascularity and tumor grade in many cancers including glioblastoma^[14,15]^.

CSCs exhibit resistance through intrinsic and acquired mechanisms against chemotherapeutic agents including temozolomide (TMZ)^[16]^. As such, novel treatments targeting this tumor subset are of paramount importance. We hypothesized that, since HIF-2α mediates the effects of hypoxia on CSCs, its silencing would decrease CSC functions and reduce the stemness within the tumor. HIF-2α silencing would presumably have the most potent effect on CSC functions under hypoxic conditions since that is when HIF-2α is overexpressed. If HIF-2α can successfully modulate the CSC phenotype, it may act in a complementary manner to chemotherapeutics such as TMZ in order to significantly reduce tumor recurrence and allow for a better survival rate.

## METHODS

### CSC derivation and characterization

Except where noted, cell culture media and supplements were purchased from Invitrogen (Carlsbad, CA). The CSCs were derived as described in the literature^[17,18]^. Briefly, serial dilutions were made of U87 cells in neural stem cell (NSC) media containing DMEM/F12 1:1 media, B27 serum-free supplement (1×), penicillin (10,000 IU/mL), streptomycin (10,000 μg/mL), 20 ng/mL fibroblast growth factor (FGF), 50 ng/mL epidermal growth factor (EGF), HEPES 1 M solution, and 5 mg/mL heparin (Sigma-Aldrich, St. Louis, MO). Dilutions were continued until neurospheres formed. CSCs were characterized based on their pluripotency, limiting dilution assays, and immunofluorescence and found to be consistent with previous results^[18,19]^. Using limiting dilution, neurospheres formed, which were capable of being passaged at least 10 times. The neurosphere formation rate was quantified, as well as their proliferative capacity. After primary spheres formed, they were dissociated and characterized. Supernatants were stored at −20 °C for use as a conditioned medium and for ELISA assays.

Primary samples obtained from patient brain tumors were obtained following institutional review boardapproved protocols (UCLA) and graded according to World Health Organization-approved guidelines. Stem cell-enriched fractions from these samples were a generous gift from Dr. Masterman-Smith. Briefly, brain tumor tissues were isolated as described^[20]^ and enriched for stem cells using serial dilutions in NSC media using the aforementioned protocol, forming brain tumor stem cells (BTSCs). Cells were seeded in a NSC growth and enrichment medium consisting of DMEM/F12 medium supplemented with 1:50 B27, 20 ng/mL FGF, 50 ng/mL EGF, 1:100 penicillin/streptomycin, and 1:100 Glutamax and 5 mg/mL heparin. Heparin, bFGF, and EGF were supplemented weekly and Glutamax bi-weekly. Neurospheres were passaged using enzymatic dissociation with TrypLE and glass pipet dissociation^[20]^.

### Differentiation of monocyte-derived macrophages

Human peripheral blood mononuclear cells were collected from healthy donor blood (Blood Center of New Jersey) that was de-identified and subsequently sorted by density gradient centrifugation using Ficoll-Hypaque density gradient (Sigma-Aldrich). Further purification was performed using CD14 microbeads (Miltenyi Biotec, Auburn, CA) as specified by the manufacturer. Monocytes were cultured for 7–10 days in RPMI 1640 supplemented with 10% FBS, 1% P/S, 4 mM *L*-glutamine, and 50 U/mL GM-CSF (R&D Systems, Minneapolis, MN). Following differentiation, cells were primed with either 1 mg/mL LPS or IL-4 (Sigma-Aldrich) for two days, resulting in M1 or M2 macrophages, respectively^[18]^.

### Chemotherapeutic effect of HIF-2α short interfering RNA

For experiments mimicking hypoxic conditions, cells were cultured with 100 μM deferoxamine mesylate (DFX) (Sigma-Aldrich), a hypoxia mimetic^[21,22]^. A Silencer Select short interfering RNA (siRNA) against human HIF-2α (Dharmacon, Lafayette, CO) was delivered to cells using two different transfection systems: (1) Lipofectamine RNAiMAX as described by the manufacturer (Invitrogen); and (2) cationic amphiphilic micelles complexed with transfection lipids as described in a previous publication^[23]^. Silencer Select Negative Control siRNA (Invitrogen) or Luciferase siRNA (Invitrogen) was used as a control, nontargeting sequence. After 24 h, 1 mM temozolomide (Invitrogen) was added to the media. After 48 h, an 3-(4,5-dimethylthiazol-2-yl)-2,5-diphenyltetrazolium bromide assay (Promega, Madison, WI) was performed to evaluate viability as a measure of chemotherapeutic response to a combined HIF-2α siRNA and temozolomide therapy.

### Western blotting

Western blots were performed using whole cell lysates from macrophages primed with either LPS or IL-4 and transfected with HIF-2α siRNA, and using cytoplasmic extracts of U87s and CSCs treated with or without 100 mM DFX for 24 h. Complete Mini EDTA-free protease inhibitor cocktail (Roche Diagnostics, Indianapolis, IN) was used to prepare whole cell lysates in RIPA buffer. Lysates were run on an 8% acrylamide gel at 100 V until the dye front passed through the stacking layer and, subsequently, at 150 V until the dye front reached the bottom of the gel. Precision Plus Protein standard (Bio-Rad, Hercules, CA) was used as a molecular weight ladder. The gel was transferred onto a nitrocellulose membrane for 1 h at 100 V and blocked in 5% BSA in TBST for 1 h. The membranes were incubated in primary antibody overnight at 4 °C on a shaker in blocking buffer. Western blotting was performed using a rabbit anti-HIF-2α antibody (ab199) (Abcam, Cambridge, UK) at 1:500 and rabbit anti-glyceraldehyde 3-phosphate dehydrogenase (GAPDH) (14C10) (Cell Signaling Technology, Danvers, MA) as the housekeeping gene. After washing three times for 10 min in TBST, blots were incubated in horseradish peroxidase-conjugated secondary antibodies for 1 h at room temperature on a shaker. After washing three times for 10 min in TBST, membranes were incubated in SuperSignal West Pico Chemiluminescent Substrate (Thermo Fisher, Waltham, MA) for 5 min and exposed to film for 5 and 10 min.

### Migration assay

Transwell filter chambers with 8 μm pores (BD Biosciences, San Jose, CA) were used in a 24-well plate for the migration assay. U87, CSCs, or BTSCs (700,000 cells/350 μL) within DMEM/F12 medium were seeded into the upper well of the insert, while the lower well contained 600 μL of LPS or IL-4 stimulated macrophage conditioned media, LPS or IL-4 supplemented macrophage media, or unconditioned macrophage media (RPMI, 10% FBS, 1% P/S, and 4 mM *L*-glutamine). Chambers were incubated at 37 °C and the cells were allowed to migrate for 24 h. The outer side of the insert was gently rinsed with PBS prior to imaging. Migrated cells were counted under a light microscope in 10 randomly chosen fields in the bottom well with 10× objective. At least 50 cells were analyzed per experiment. All other co-culture experiments were performed using 4 μm pore size transwell chambers (BD Biosciences).

### Immunofluorescence of spheroids

Spheroids of U87 cells were grown for three days using the hanging drop method. Each droplet contained 20,000 cells in 20 μL medium. Each spheroid was plated into a well of a 96-well plate containing 50 μL of 2% agarose in PBS after waiting 5 min for the gel to solidify. The wells were filled with 100 μL media. After 24 h, the spheroids became smaller and tightly packed. Neurospheres of CSCs were prepared by culturing in NSC media, as described above. Spheroids were cryosectioned in 20 μm slices, stained, and then imaged by confocal microscopy in Lab-tek chambers (Thermo Fisher). Cells were stained with antibodies for Nestin (ab6320) (Abcam) and CD133 (PAB12663) (Abcam) for neural stem cell markers. Antibodies to glial fibrillary acidic protein (BT-575) (Biomedical Technologies Inc., Mt. Arlington, NJ), βIII-tubulin (Sigma-Aldrich), and myelin basic protein (Abcam) were used as markers of differentiated cells. DAPI (Invitrogen) was the nuclear stain used.

### Statistics

The data are presented as means ± standard error of the mean (SEM). Each experiment was repeated three times unless indicated otherwise, and comparisons were made using one-way ANOVA and post-hoc analysis, as indicated in the figures.

## RESULTS

**Figure 1.**
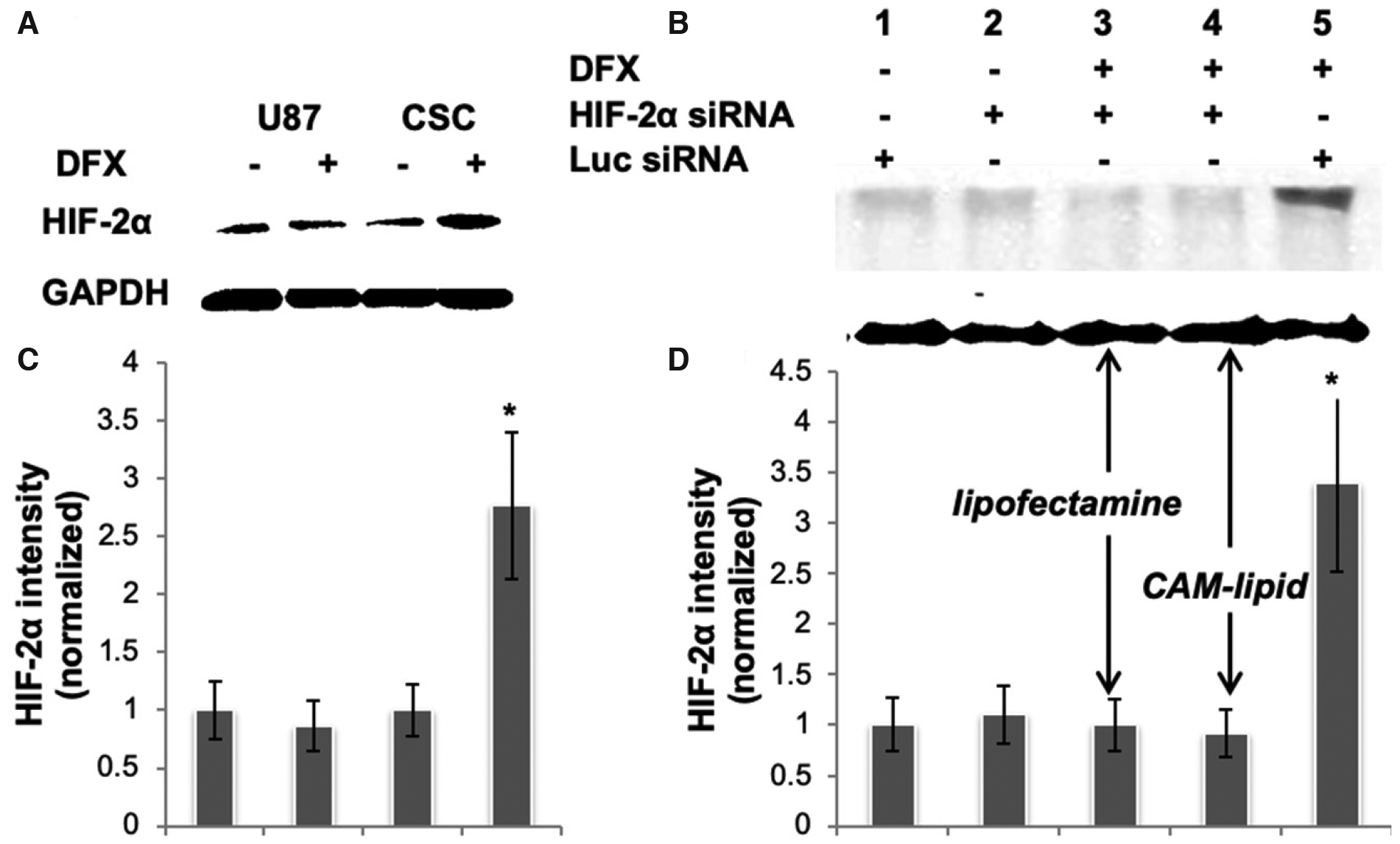
A, B: HIF-2α expression in CSCs. Representative immunoblots of whole cell lysates are shown. Blots were probed for HIF-2α, and GAPDH was used as a housekeeping gene to normalize for protein loading. C, D: bar graphs obtained by densitometric analysis of Western blot data are shown. Results (mean ± SEM) represent the ratio between HIF-2α and GAPDH levels and are further normalized to the conditions shown in Lane 1 of the corresponding blot; *P* < 0.05, n = 2 independent experiments. Lanes 3 and 4 in (B, D) received the same HIF-2α siRNA but with different transfection reagents, showing that the CAM lipid is at least as effective as Lipofectamine RNAiMax in this application. siRNA: short interfering RNA; HIF: hypoxia inducible factor; CSC: cancer stem cell; DFX: deferoxamine mesylate; CAM: cationic amphiphilic macromolecule; GAPDH: glyceraldehyde 3-phosphate dehydrogenase

We first sought to establish a tractable cell culture model that reflects HIF signaling in glioma cancer stem cells. The protein expression levels of HIF-2α in U87 glioma cells and in U87-derived CSCs under hypoxia and normoxia conditions were evaluated [Figure 1A], where hypoxia was mimicked using the chemical inducer DFX. In this hypoxia-mimetic environment, the CSC population is enriched and overexpresses HIF-2α, while levels under normoxic culture conditions are low for both U87 and CSC cells. Quantification of bands and normalization to GAPDH revealed that CSCs treated with DFX expressed nearly 300% of the HIF-2α levels as compared to control [Figure 1C]. The increase in HIF-2α expressed by DFX-treated CSCs was silenced effectively using a siRNA specific for HIF-2α and delivered with either the commercial transfection reagent, Lipofectamine RNAiMAX, or with a cationic amphiphilic macromolecule (CAM) lipid formulation, which we developed previously and showed to be at least as effective as siRNA-based gene silencing in U87 cells^[23]^ [Figure 1B and D].

**Figure 2.**
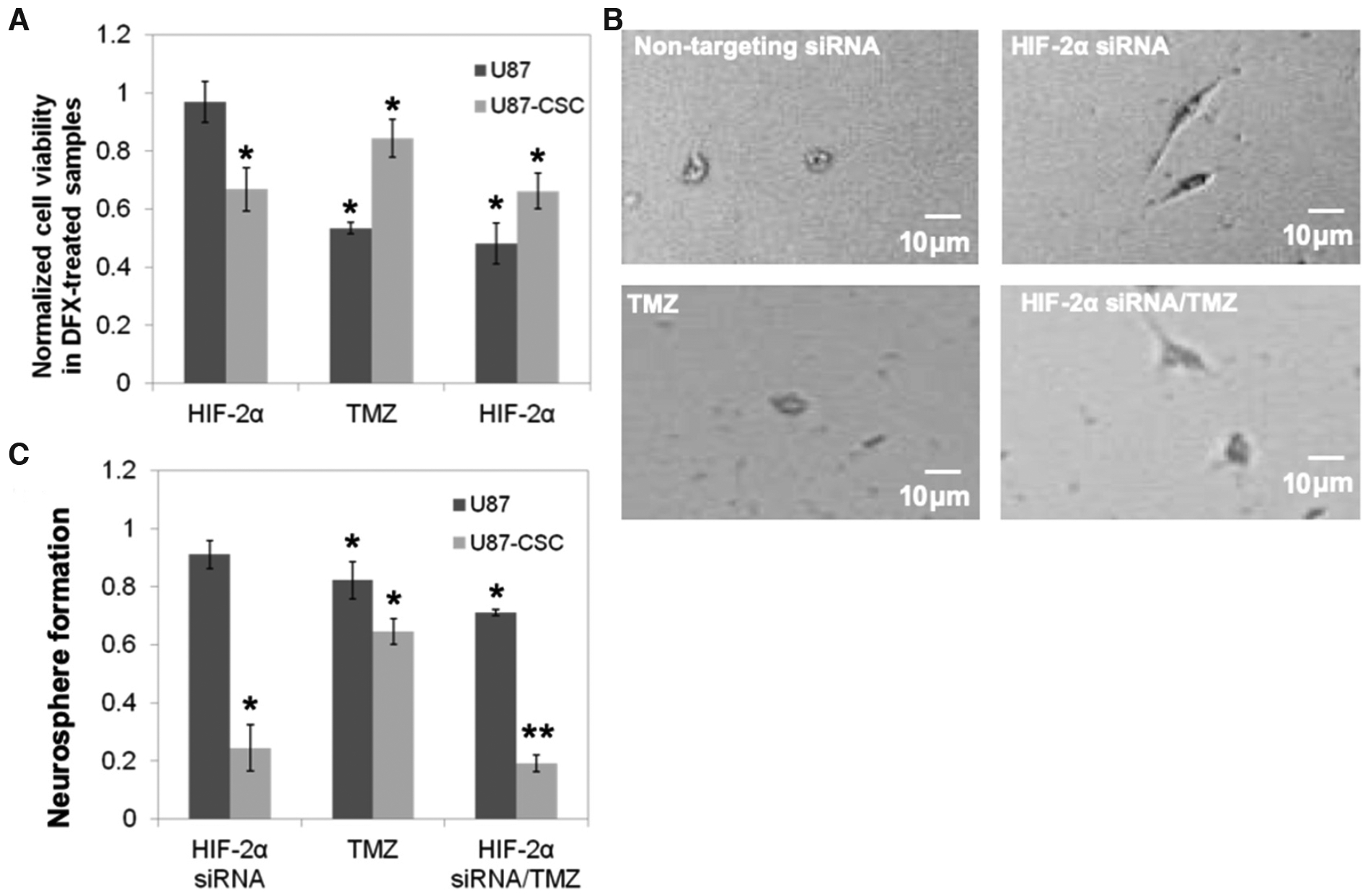
HIF-2α silencing reduces chemoresistance of CSCs. A: viability was assessed of HIF-2α silenced or a non-targeting control siRNA treated U87-CSCs at 48 h following incubation with the hypoxia mimetic, DFX, and the DNA alkylating agent TMZ. Values were normalized to a control of non-targeting siRNA and no TMZ, which is also the comparison group for statistics; *P < 0.05, *n* = 3. B: phase contrast microscopy of CSCs treated with non-targeting or HIF-2α siRNA and/or TMZ treated depicts an altered morphology upon HIF-2α siRNA treatment. C: neurosphere formation in the presence of DFX after seven days following a 48-h treatment with HIF-2α and/or TMZ. Data were normalized to cells treated with a non-targeting control siRNA, which is also the comparison group for statistics; *P < 0.05, **P < 0.01, *n* = 3. siRNA: short interfering RNA; HIF: hypoxia inducible factor; CSC: cancer stem cell; DFX: deferoxamine mesylate; TMZ: temozolomide

Having established the DFX model of hypoxia and an effective HIF-2α silencing protocol, we next examined the effect of HIF-2α expression on responsiveness to the gold standard chemotherapy for glioblastoma, the alkylating agent TMZ. Previous studies have found that the responsiveness of CSCs to TMZ depends on a number of factors including the O-6 methylguanine DNA methyltransferase status of the cells, dosing scheme, and presence of hypoxia^[24,25]^. U87 or CSCs, each in the presence of DFX, were treated with TMZ and/or HIF-2α siRNA. At a concentration of 1 mM, TMZ exerts a strong cytotoxic effect on the viability of U87 cells, but the CSC sub-population is only slightly reduced by this concentration of TMZ [Figure 2A]. HIF-2α silencing alone slightly decreases the viability of CSCs and has an additive effect with TMZ to produce a somewhat greater reduction in viability under the conditions studied. We observed by phase contrast microscopy that CSCs treated with HIF-2α siRNA exhibited a morphology consistent with greater cell spreading, which is often indicative of a more differentiated state, than their HIF-2α expressing counterparts [Figure 2B]. Phenotypically, this is reflected in the 80% decrease in neurosphere formation of HIF-2α silenced CSCs with or without administration of TMZ [Figure 2C]. Together, these observations point to a significant role for HIF-2α in mediating CSC stemness.

**Figure 3.**
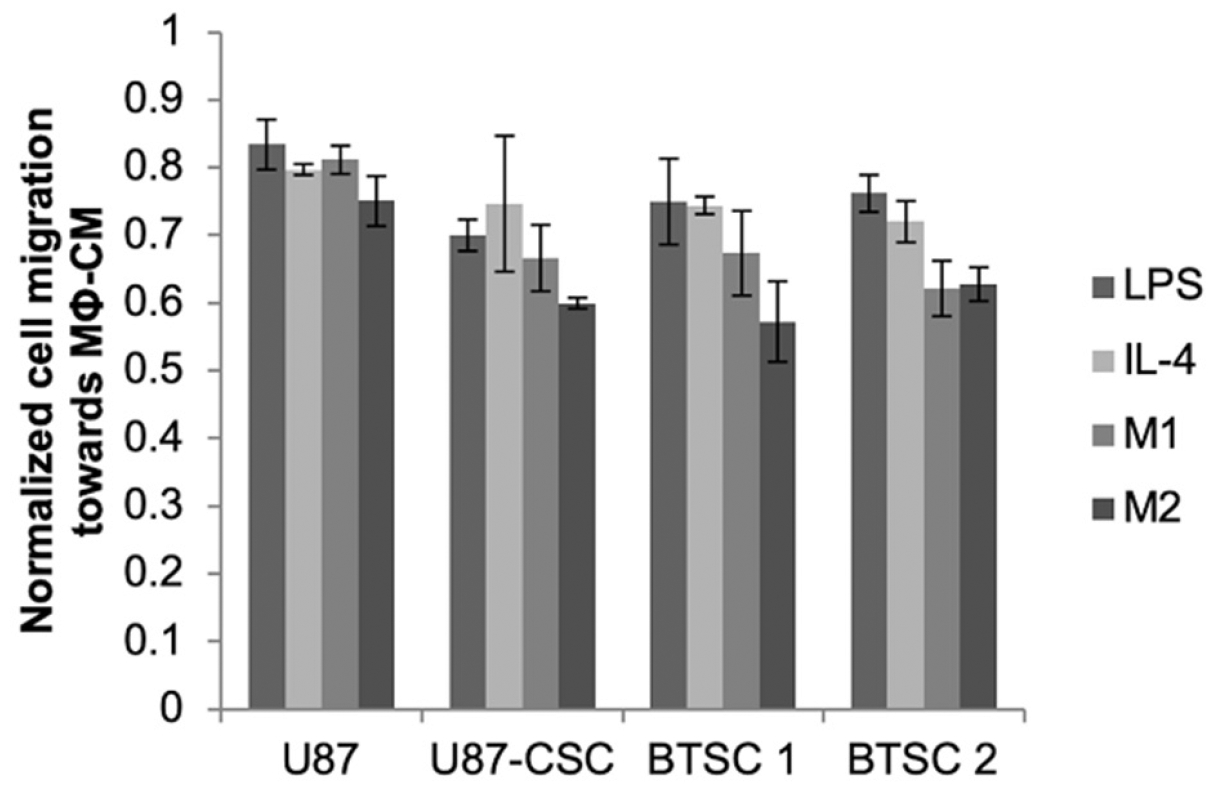
HIF-2α silencing reduces migration rates of CSCs derived from either U87 cells or BTSC. Cells grown in the presence of DFX were treated with HIF-2α or non-targeting siRNA for 24 h, at which time they were seeded into the top well of a Transwell system and allowed to migrate for 24 h towards medium conditioned by M1 or M2 macrophages or supplemented with LPS or IL-4 as indicated. At least 50 cells per field and 10 fields were counted (10×) of cells that migrated through the Transwell. Normalized cell migration was calculated as the fold increase of cell migration over non-targeting siRNA-treated cells exposed to the same medium conditions. Statistics compare each of the respective medium conditions to U87 cells; *P < 0.05, *n* = 3. BTSC: brain tumor-derived stem cell; siRNA: short interfering RNA; HIF: hypoxia inducible factor; CSC: cancer stem cell; DFX: deferoxamine mesylate; LPS: lipopolysaccharide; IL-4: interleukin-4

Glioma cancer stem cells receive cues from other cell types, such as macrophages, present within the tumor milieu. We previously found that exposure of CSCs, but not parental U87s, to macrophage-conditioned medium stimulated their migratory capacity^[18]^. The magnitude of the effect varied depending on whether medium was conditioned by M1- or M2-polarized macrophages or merely with LPS or IL-4, with the greatest effect observed with media conditioned by M2-polarized macrophages. This increase was modulated by silencing of HIF-2α [Figure 3]. For each cell type (parental U87, U87-CSC, or brain tumor isolated CSCs) and conditioned medium type, the migration rate was reduced after HIF-2α silencing, but again the magnitude of the effect was greater for CSCs compared to parental U87s and was statistically significant only when the medium was conditioned by the M2 macrophages, which are associated with the promotion of malignant phenotypes in cancer cells.

**Figure 4.**
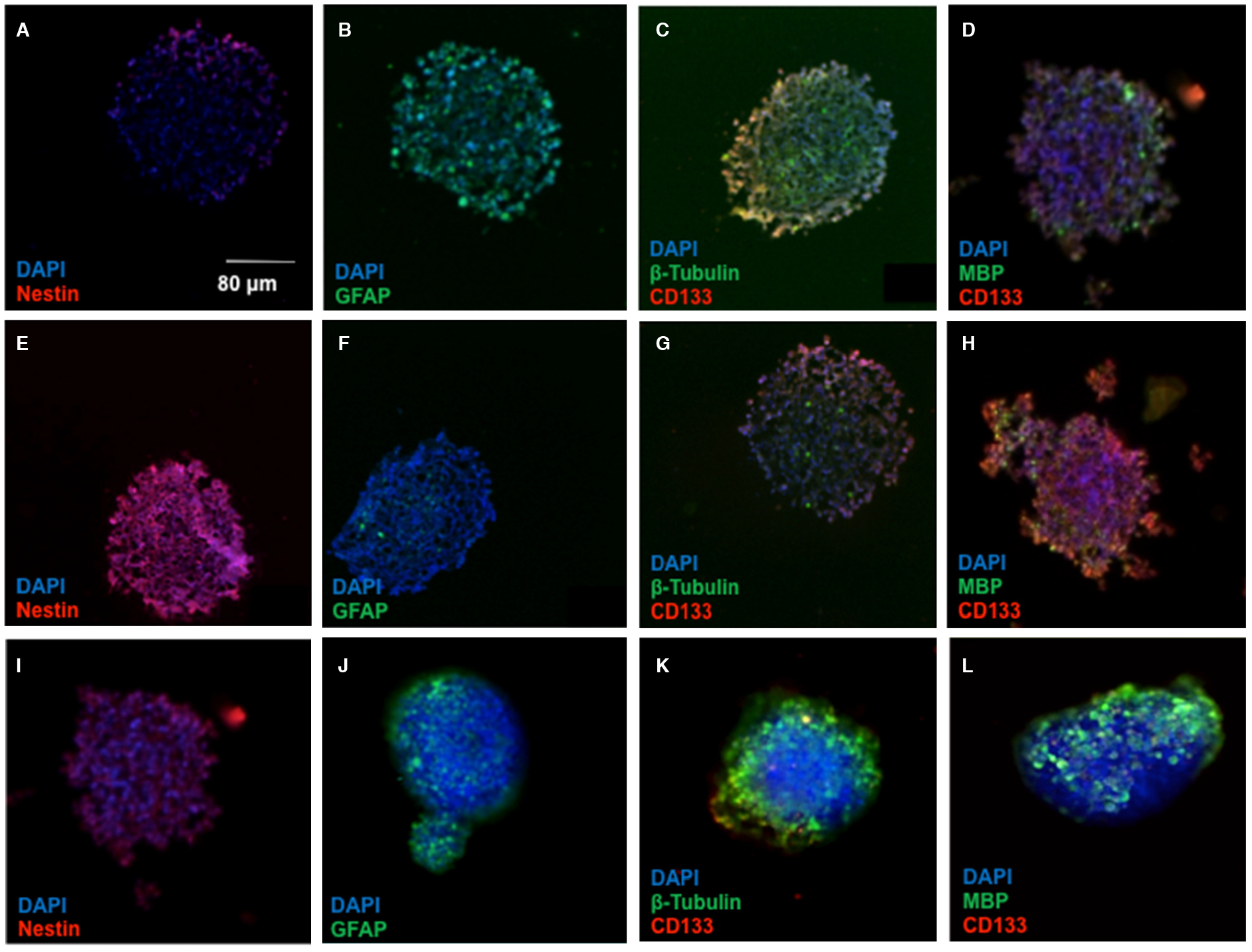
HIF-2α silenced CSC neurospheres display differentiation markers. The micrographs depict cryosections of: U87 spheroids (A-D); CSC neurospheres (E-H); and HIF-2α silenced spheroids (I-L). Neurospheres of all categories were stained for stem and differentiated cell markers as indicated. Cells were stained with antibodies to nestin and CD133 for neural stem cell markers, and antibody stains of GFAP, β-tubulin, and MBP were used as markers of differentiated cells. DAPI was the nuclear stain used. HIF: hypoxia inducible factor; CSC: cancer stem cell; GFAP: glial fibrillary acid protein; MBP: myelin basic protein; DAPI: 4’,6-diamidino-2-phenylindole

The spread morphology and decreased neurosphere formation in HIF-2α silenced CSCs suggests an alteration in the “stemness” of the CSCs. To provide some mechanistic insight into these phenotypic changes, we examined stem and differentiation markers in spheroids treated with HIF-2a siRNA. Spheroids treated with the hypoxia mimetic, DFX, and HIF-2α siRNA were stained with antibodies to several stem cell and differentiation markers [Figure 4]. In contrast to parental U87s, neurosphere CSCs exhibited significant staining for nestin and CD133, which are both neural stem cell markers, while showing limited expression of glial fibrillary acid protein, β-tubulin, and myelin basic protein, all markers of differentiated cells. Notably, the CSCs treated with HIF-2α siRNA exhibited greatly reduced staining of nestin and CD133, accompanied by an increased expression of all three differentiated cell markers. This further confirms our result that HIF-2α silencing strongly induces differentiation, suggesting an additional mechanism by which HIF-2α inhibition could ameliorate the malignant phenotype in glioma.

## DISCUSSION

Gene silencing is an essential tool for dissecting the role of individual gene products in a biological process and, with a number of recent antisense and siRNA drug approvals by the United States FDA, rapidly becoming a significant therapeutic modality. To probe the role of HIF-2 in maintaining the properties of glioma cancer stem cells, we established a cell culture model where we could induce HIF-2α chemically and silence it via efficient siRNA delivery. DFX is an iron chelator that has been characterized extensively as an inducer of HIF activity in multiple settings, including vascular development, wound healing, and cancer. Induction of HIFs by DFX in glioblastoma cells has been shown to affect their differentiation status as well as their migration rates^[21,22]^. As in previous studies on glioma stem cells^[26]^, in this work, DFX was used to induce HIF-2α expression. Robust silencing was achieved comparably using either our CAM-based delivery vehicle^[23,27]^ or Lipofectamine for transfection. The CAM system, which utilizes a pH-sensitive mixture of lipids and amphiphilic polymer to promote endosomal escape^[28]^, produced efficient HIF-2α silencing in hypoxic CSCs [Figure 1].

Hypoxia has long been recognized as a characteristic of solid tumors, and HIFs have been identified as transcriptional effectors that coordinate adaptive cellular responses, e.g., in metabolism^[29]^. While greater focus has been given to HIF-1α^[30]^, recent findings have indicated that HIF-2α is preferentially expressed in glioma cancer stem cells and correlates negatively with patient survival in clinical glioblastoma^[7,31]^. Recent studies have shown that short hairpin RNA knockdown of HIF-2α induces apoptosis, reduces cell growth, inhibits angiogenesis, and diminishes neurosphere formation and conversion of glioma cells to glioma stem cells following temozolomide treatment^[26,32]^. Moreover, knockdown of HIF-2α has been shown to inhibit growth of tumors from CSCs in mice^[26]^. By silencing HIF-2α with siRNA, we observed a multitude of phenotypic effects that were exclusive or preferential to CSCs derived from the U87 glioma cell line and that were not present in the parental cells. These include inhibition of neurosphere formation, adoption of a spread morphology in dispersed culture, and a reduction in cellular migration [Figures 2 and 3]. Thus, silencing hypoxic mediators where these cells reside or disrupting the signaling between macrophages and CSCs may have therapeutic potential.

The changes in cellular phenotype and neurosphere “tissue” formation were reflected in changes in key differentiation markers. Markers of stemness, which are upregulated in CSCs relative to parental U87s, are diminished in HIF-2α silenced spheroids, while markers associated with neural and glial cells were restored [Figure 4]. Taken together, these results suggest an essential role for HIF-2α activation in promoting CSC development. Indeed, a clinical trial is underway utilizing a small molecule HIF-2α inhibitor to teat glioblastoma^[7]^. Furthermore, as our results indicate a modest additive effect with temozolomide in cell killing and complementary influences on CSC differentiation status, combination therapies that combine chemotherapeutics with HIF-2α silencing or inhibition are an attractive avenue in the pursuit of a therapy that can eradicate the CSCs that drive recurrence of malignant cancers such as glioblastoma^[33,34]^.
